# Lateral Multimodal Learning in a Saudi EFL Context: Investigating the Perceptions of Teachers and M2E Female Learners

**DOI:** 10.12688/f1000research.109454.1

**Published:** 2022-03-30

**Authors:** Arif Ahmed Mohammed Hassan Al-Ahdal, Fahd Hamad Alqasham, Mohammed Ali Mohammed Qarabesh

**Affiliations:** 1 Department of English Language, College of Arts and Sciences, Methnab, Qassim University, Saudi Arabia

**Keywords:** COVID-19 Epidemic, Distant Education, e-learning, English as a Second Language (EFL), English Language Teaching (ELT), Remote Education, Saudi University Students and Teachers

## Abstract

**Background: **ELT scenario in Saudi Arabia has undergone a sea change since the pandemic. With an aim to maximize resource utilization and ensure wide learner base, college students (male and female) are taught simultaneously, the former in a face-to-face mode and the latter in an audio-only mode. The nomenclature given to this unique classroom design by the researchers is Lateral Multimodal Learning (LML), one which has its own advantages and disadvantages. This mode of learning puts a great deal of pressure on the teachers as they must attend to a huge number of students with different needs and levels of competence, whereas it ensures best utilization of infrastructural and human resources by the administrations. Being a newly developed educational model, it is important to assess the efficiency of this type of learning. **Methods** **:** This study evaluates the model from the point of view of students (99), using a questionnaire, and that of teachers (06), using semi-structured interviews. **Results** **:** The results show that Saudi female students present high perceptions of learning via LML (M=4.03); are satisfied with this type of learning (M= 3.81) and the aids applied in learning via LML (M= 4.02). Findings also show moderate perceptions on the difficulties they encountered while emerging in LML mode (M =3.39). Furthermore, the study shows correlation between the four domains, i.e., perceptions, satisfactions, challenges, and aid. The highest correlations were between perceptions and satisfactions (r=.719); perceptions and aids (r=.659), and satisfaction and aids (r=.656). The teachers’ interviews show their agreement on the efficacy of LML as being professionally fulfilling and one that they would like to continue with in the future too. **Conclusions: **The study concludes with recommendations, which would be of great benefit and help for all parties or stakeholders involved.

## Introduction

Lateral Multimodal Learning (LML) is a pedagogical concept that took shape in the unique educational circumstances presented by the novel coronavirus (COVID-19) pandemic to the Saudi teaching community. Given the nature of gender segregated classrooms in the country, teachers (whether male or female) are wont to teach multimodally in lateral classrooms. The design is that learner groups are still gendered but each learns with the same teacher, who teaches one group F2F and the other remotely in an audio-only mode. To fulfil the social and cultural restrictions, only male students can talk to and see the teacher; whereas female students can only talk to, but not seeing the teacher, nor can the teacher see them. The global society was severely impacted by COVID-19 pandemic, which brought the world to a standstill in 2020. Educators worldwide were forced to migrate to online learning in such unusual circumstances. In-person instruction posed an unacceptable danger of getting and transmitting the illness. This abrupt and unexpected transition brought unforeseeable ramifications to educators, as the globe continues to see its impacts. In this background, the current study is guided by the following research questions:

1. What perceptions do Saudi EFL language instructors have about LML?
2. What perceptions do Saudi EFL female students have about LML?
3. Are Saudi EFL female students satisfied with LML Learning mode?
4. Are there any correlations between EFL Saudi female students' perceptions, satisfaction, difficulties and aids toward LML mode?
5. Do Saudi EFL female students get help from other aids while learning though LML?

These topics are addressed via an examination of the most recent research released in the fields of EFL and language instruction after the pandemic hit in 2020. The issue was investigated in available literature using search terms such as ‘English language’, ‘Saudi universities’, ‘COVID-19’, and ‘EFL’. The next parts examine Saudi Arabia’s reaction to the epidemic, how schools and colleges continued to educate students after closures, and prior research on e-learning in the Saudi context. Following that, the most significant problems faced by Saudi university EFL professors and students in online learning are discussed, as well as the positive consequences noticed as a result of the transition to online learning. Many studies were conducted regarding Covid-19 (Al-Ahdal & Alqasham, 2020; Hazaea *et al.*, 2021; Avelar *et al.*, 2019; Sefara *et al.*, 2019; Vanslambrouck *et al.*, 2019; Vattøy & Smith, 2019) around the globe and in Saudi. All the previously mentioned studies either focused on the challenges that instructors faced while teaching online (Al-Ahdal, & Alqasham, 2020; Hazaea *et al.*, 2021) or students’ attitudes towards online learning, or even the psychological statues of the learners ( Truong & Wang, 2019; Tsai, 2019). To the best knowledge of the researchers, no previous research nationally or internationally evaluates the LML mode. Hence, this study is an endeavor to conceptualize and determine the boundaries of this new mode of learning.

## Literature review

LML is a new mode of teaching, which stands for Lateral Multimodal Learning, *i.e.*, a classroom situation in which a teacher teaches male and female students simultaneously in segregated classrooms, the former being face-to-face and the latter being remote. National and cultural sentiments are honored in this model by restricting the interaction of female learners to a strictly audio mode.

### Saudi response to the pandemic

Since the outbreak of the pandemic, the Kingdom of Saudi Arabia (KSA) has been vocal in its humanitarian message. That is, regardless of color, gender, religion, or nationality, human life is more valued than everything else. As a result, throughout the first few days of March 2020, an emergency plan saw the closure of everything that threatened to jeopardize people's health and well-being, including schools, universities, and public and private organizations ( Avelar *et al.*, 2019).

However, the country’s high ambitions and aspirations were not harmed by these physical closures. On the contrary, it exacerbated them. As a result of the closures, all Saudi sectors, including education, embraced digital technology to assist sustain services while limiting the disease ( Aylett *et al.*, 2021). Within a short period of time, the whole nation started transitioning to remote learning settings, whether via television broadcasts or communication through different online platforms: Telegram, Zoom, Teams, WebEx, and Blackboard. However, during the start of the epidemic, the situation was far from optimal. Many students and instructors lacked the digital literacy and internet capacity required to fully utilize these facilities. Despite this, they rose to the occasion and eventually adapted to the new educational standard.

### Academic institutions in KSA

The Saudi Ministry of Education established *Madrasati* (meaning ‘my school’), a national Digital Teaching Platform (DTP), in the 2020-2021 academic school year. The objective was to establish a centralized platform for providing online teaching to nearly six million students enrolled in Saudi public institutions from kindergarten to twelfth grade ( Canals & Al-Rawashdeh, 2019). With the start of the new school year, instructors started to regain lost learning. Middle and high-school students took online lessons during designated morning hours, while primary school students had afternoon sessions. These efforts guaranteed that each level received an appropriate amount of learning time and helped alleviate internet traffic congestion caused by a large number of concurrent users. The afternoon hours allotted for elementary school kids enabled parents to help their young children in attending their online lessons and to assist them with their learning ( Canals & Al-Rawashdeh, 2019).

On the other hand, since most Saudi institutions had previously integrated digital communication and learning technologies though on a different scale, they were inherently better equipped to migrate to an online learning environment. For example, university students are provided with an official email address upon enrollment. As a result, contact between students and their institutions was not disrupted. Additionally, most Saudi institutions used Blackboard®, a Learning Management System (LMS) that supports both synchronous and asynchronous forms of instruction. However, before the pandemic, this software was not widely utilized and functioned just as a complement, and its e-learning customers are still discovering its benefits.

### E-learning of English in Saudi Arabia

Numerous phrases are often used when addressing the use of technology to offer educational materials, including e-learning, online learning, electronic learning, digital learning, and technology-enhanced learning. All these terms refer to ‘a collection of technology-mediated instructional approaches that may be utilized to promote student learning and include features of evaluation, tutoring, and teaching’ ( Chan, 2021). In other words, digital and online technologies are the main and sole means of providing educational information and teaching. This may be synchronous ( *i.e.*, teaching happens in real time with both students and teachers present online) or asynchronous ( *i.e.*, the content and instruction are available for the students to access any time via recordings of the lessons or independent online activities).

A vast amount of prior research has examined the efficacy of integrating technology into EFL instruction at KSA colleges. However, these studies used technology tools in conjunction with face-to-face teaching, a process known as blended learning. Blended learning is described as ‘any formal education program in which a student learns at least in part via online learning’. That is, just a portion of the instructions are sent online; the remainder are delivered in person. This strategy has been demonstrated to be effective in learning English in the Kingdom of Saudi Arabia. Additionally, Correa-Baena *et al.* (2018) discovered that supplementing in-person teaching with online activities ( *i.e.*, viewing lesson-related videos and engaging in discussion forums) enhanced university EFL learners’ listening and speaking abilities. Other research examining university EFL students’ evaluations of the blended learning model’s efficacy in acquiring English reveal favorable sentiments regarding this strategy ( Fu *et al.*, 2019; Hackl & Ermolina, 2019; Hux *et al.*, 2021; Hwang *et al.*, 2019).

Nonetheless, these studies look at technology as a complement to face-to-face training, not as a substitute, as was the situation during the epidemic. The blended learning paradigm is distinguished from the e-learning model by the fact that the former includes some in-person teaching, whilst the latter is totally online, rendering the outcomes incomparable and warranting separate discussion.

### Difficulties in online English teaching during the pandemic

Obstacles and hindrances in English language teaching (ELT) that existed before the COVID-19 epidemic were worsened by the abrupt change to online schooling and the resulting mandatory adaptations. One of the issues most often addressed in ELT is the motivation of language learners to learn ( Lau & Gardner, 2019). Language instructors in Saudi Arabia have often noted a lack of students’ motivation ( Macalister & Nation, 2019), which is most likely due to students’ poor competency ( Marcoux *et al.*, 2021), but may also be due to other causes ( Mira & Fatimah, 2020). According to previous research, online learning environments are more engaging and favorable to raising students’ motivation ( Nakayama, 2018). However, distant learning is ‘situationally sensitive’ ( Newton & Nation, 2020), impacted by external variables such as time restrictions, grades, and the learning environment of students. The epidemic exacerbates these problems. Indeed, a recent study of over 1,000 academic English teachers in 99 countries found that one of the respondents’ primary worries during the epidemic was student motivation ( Prakash & Murthy, 2019).

According to a study by Rayganand Moradkhani (2020), 68 percent of respondents stated that students are less motivated to learn online during the COVID-19 epidemic. This demotivation is not always attributable to the online learning environment. According to other sources, the major reasons of student demotivation during the pandemic include being socially isolated, having a slow internet connection, dealing with distractions at home, and being unable to meet class goals ( Schmid *et al.*, 2020). Since practically all KSA education is now delivered online, students are required to attend class from their homes, where they may feel isolated from their classmates or distracted by siblings and other family members who are also studying or working remotely. Only those who need practical training or laboratory work continue to engage in face-to-face instruction.

A further factor for students’ demotivation might be mental health concerns, considering the strong correlation between motivation and anxiety ( Sefara *et al.*, 2019). Numerous studies found that many students had anxiety, sadness, and post-traumatic stress disorder as a result of the epidemic, particularly during its first stages ( Truong & Wang, 2019; Tsai, 2019). Learning during the COVID-19 pandemic may be very difficult for kids owing to the rapid changes taking place around them and their fears about the sickness ( Vanslambrouck *et al.*, 2019; Vattøy & Smith, 2019). Consequently, students may experience psychological anguish, as a result of their anxiety of being unable to continue their academic advancement ( Villegas *et al.*, 2020). While students and instructors have continued the educational process, they are doing so under less-than-optimal settings and face multiple obstacles, all of which may influence motivation.

Another challenge raised by the 2020 pandemic is digital preparedness. Digital readiness may be described as ‘the degree to which individuals succeed or struggle while attempting to navigate their surroundings, solve issues, and make choices’ via the use of technology ( Wang, 2019). The transition to remote learning surprised the globe and left many students and educators unprepared for the new duties they were required to undertake. Wilson *et al.* (2020) conducted a Blackboard readiness assessment with 25 students enrolled in Taif University’s English language department. The results implied that students are digitally unprepared and lack technical abilities. Additionally, the author discussed how she trained her pupils to download and annotate PDF files, as well as demonstrating the importance of assisting students through tough moments. Indeed, in many situations, English language teachers have been forced to assume the job of a technical support expert, instructing students on how to download, upload, and distribute their work, among other things. Those with lesser English proficiency levels, particularly when working with language learners, may have extra challenges using digital technology, given that majority of technology use is offered in English ( Yasuda *et al.*, 2021).

Additionally, students may only access instructional information and online courses if they have access to adequate equipment and a dependable internet connection (on both the students’ and instructors’ end). Thus, internet connectivity and bandwidth have a substantial impact on learners’ online experiences, since their learning experiences are contingent on the dependability of their internet connection ( Monteiro *et al.*, 2020). Fandiño *et al.*, (2019) who conductedqualitative interviews with 12 EFL university students at King Saud University found that students had technical difficulties when taking lessons on Blackboard, ranging from incompatible equipment to audio interruptions to being locked out by the site. These difficulties become more prevalent at specific periods of the day as a result of higher internet traffic using the platform concurrently. Similarly, Vattøy (2020) studied the effects of online assessment on 20 EFL students at Onaiza institutions and reported that 64.3 percent of respondents experienced ‘frequent disconnections’ that harmed their learning and online assessments. Interruptions caused by a sluggish or insufficient internet connection degrade the quality of the online learning experience. They may also have a detrimental effect on learning, since they may result in student dissatisfaction and demotivation ( Jiang, 2018).

Additionally, there is a problem with inadequate technical help. Due to the high volume of students and professors requiring assistance at any one moment, students do not get assistance when they are in need ( Jansen *et al.*, 2021). Additionally, students with impairments encounter accessibility challenges and may not get the necessary help when they visit their educational institutions in person. For instance, persons with visual impairments may have difficulty seeing the digital whiteboard, reading instructor-posted material, or engaging in classroom conversation. They need access to voice-to-text software, which, at the time of writing, does not support all digital platforms and file types. Similarly, students who are deaf or hard of hearing are not always able to access closed captioning or subtitles for oral or video courses. These accessibility concerns for students with disabilities exclude them and prohibit them from fully using online education ( Butler & Le, 2018).

It is worth noting that one of the most amazing accomplishments of the Saudi Ministry of Education in terms of general education was the creation of 23 educational television channels dubbed iEn for individuals without access to the internet. These channels feature translations in Saudi sign language for deaf pupils. Additionally, they have established three channels for students enrolled in special education. These instructional adjustments assist in mitigating some of the accessibility difficulties associated with distance education for students with disabilities. Nonetheless, such services are lacking at the university level.

On the other hand, EFL learners face substantial challenges due to a lack of visual input during online learning. Due to cultural limits and a desire to protect users’ privacy, students and instructors are not needed to switch on cameras in the virtual classroom, particularly at the tertiary level. However, ELT theory and research support the relevance of non-verbal information and facial movements in the growth of language learners. Visual cues are vital for conveying critical information that communicates the message’s overall meaning (Mackay, 2019). Thus, in an online language class, the listener’s lack of visual feedback from the speaker is a disadvantage for EFL students. As a result, several studies on online learning during the epidemic revealed that in the Saudi EFL setting, face-to-face communication with peers and instructors is preferred ( Bernstein &Woosnam, 2019). Indeed, participants in Mackay’s (2019) qualitative focus groups said that they preferred in-person sessions since they ‘lacked eye contact with the lecturer’.

Assessment is another issue when ELT is conducted remotely for a variety of reasons (Al-Ahdal& Alqasham, 2020; Hazaea *et al.*, 2021). To begin with, many language teachers have the challenge of ensuring that the work submitted by students is their own. Despite the widespread availability of anti-cheating software and plagiarism detection technologies, not all teachers are well educated to use them, and students circumvent them using a variety of academically dishonest tactics.

Vattøy (2020) observed that one of the challenges in online English instruction is the ‘ease with which the exam material may be penetrated’. They discuss numerous strategies for preventing cheating, including utilizing a plagiarism checker to verify students’ written responses, rewriting the substance of objective type questions, showing single questions in random order, and shortening the test’s allocated time. Nonetheless, there is no assurance that the student is completing the evaluation on his or her own without being physically present or using surveillance software (which would breach students’ privacy). Additionally, it complicates the process of evaluating pupils’ academic development. Mackay (2019), for example, discovered that students utilized a second device during tests to do internet searches or simply copy and paste results from other sources. Remote evaluations do not adequately portray students’ growth as a result of these evaluation difficulties.

Additionally, online learning and teaching are more time-consuming than in-person sessions, adding another layer of difficulty. Jansen *et al.* (2021) discovered that just 10 percent of English language instructors spent most of their teaching time online prior to the epidemic. However, 55 percent of instructors polled now spend 100 percent of their instructional time online. Besides teaching online, language teachers prepare content, produce materials, send and respond to emails, post tasks for students, and grade students’ work online, to mention a few of their new duties. Consequently, language instructors spend 10 to 12 hours every day in front of a computer screen ( Canals & Al-Rawashdeh, 2019). Similarly, in Jansen *et al.* (2021), in a poll of Saudi EFL university students, 85.7 percent said that preparing for online lectures takes them longer than preparing for in-person sessions. It is also troubling since, as Vattøy (2020) indicates, increased time spent online, particularly on mobile devices, is strongly associated with academic procrastination and social media addiction. Additionally, prolonged screen time and online interactions leave many users fatigued and depleted, a condition dubbed Zoom Fatigue ( Avelar *et al.*, 2019).

Additionally, there are concerns with learning systems. Apart from Blackboard LMS, students and teachers are wont to use various programs and technologies, although these have raised concerns about privacy and security. Among these is WhatsApp, a smartphone application that enables users to share text messages, photographs, videos, voice notes, and even make free phone calls to family and friends. According to Correa-Baena *et al.* (2018), WhatsApp is one of the most popular applications among university EFL students, who see the service as a tool for improving their reading, writing, and grammatical skills. Al-Ahdal and Hussein (2020) studied the use of WhatsApp as a learning tool for developing Saudi EFL learners’ wiring skills. Similarly, Ali and Bin-Hady, (2019) found that WhatsApp reduced Saudi EFL learners’ stress and helps them to overcome the anxiety associated with language learning. Al-Ahdal and Alqasham (2020) explored EFL Saudi instructors’ use of WhatsApp in classroom. However, several privacy issues have surrounded the application due to the application’s usage and sharing of users’ data.

Zoom, a videoconferencing program, had the same issues. Prior to the COVID-19 epidemic, nearly no one used Zoom for teaching, but as schools became virtual, almost everyone did. Zoom’s teacher-friendly features include live audio and video conferencing, a digital whiteboard, screen sharing, and the ability to upload teaching resources. It was also beneficial to university students. Bernstein and Woosnam (2019) discovered a favorable association between Saudi EFL university students’ Zoom usage, their assessed usefulness and acceptability of the technology. Nonetheless, security flaws were uncovered during a series of Zoombombings, in which an uninvited participant enters a Zoom session with the potential for overt disruption. Another issue was Deepfakes, which is defined as ‘the imposition of another person's face onto another person's body in video format using Artificial Intelligence algorithms’. Therefore, the majority of Saudi institutions have advised their teachers against using Zoom for teaching and online conferencing and have asked them to switch to a more secure option, such as Blackboard LMS, even if it lacks some of the useful features offered by Zoom.

### The beneficial effects of online education during COVID-19

Despite the disadvantages of online English training, some beneficial features of this have emerged. One of the most major benefits of online education is the flexibility it provides. Remote learning enables students and instructors to attend courses from any location and at any time (if learning asynchronously). Additionally, it enables students and teachers to choose from a variety of devices and applications. The study of Avelar *et al.* (2019) with Saudi university students indicated that they are more likely to utilize their mobile phones in e-learning settings, which makes accessing their classes and learning materials simpler. Additionally, the authors said that virtual learning environments allow learners to study the course whenever and wherever they are. Chan (2021), for example, noted that EFL students attended classes while at work, in the automobile, sat with family, or from the comfort of their bed, even though this is not always desired. Additionally, this flexibility saved students time spent travelling to and from their academic campuses ( Bernstein & Woosnam, 2019). Surprisingly, even teachers trapped across borders—as a result of quarantine regulations and air travel restrictions—could teach from nations separated by many time zones ( Aylett *et al.*, 2021). Thus, online education during the pandemic alleviated physical restrictions of distance and time, opening the door for a new approach to learning that is not constrained by these shortcomings.

Another advantage noted because of the shift to online English training is that students who were previously too timid to speak out in front of a big class may now be more inclined to do so in front of a screen from the comfort of their homes. For example, Villegas *et al.* (2020) studied 311 EFL university students from five major Saudi institutions and discovered that attending lessons through Blackboard (rather than in-person) assisted learners in overcoming their shyness in class discussions. Similarly, it has been said that online language learning increases introverted students' confidence in class and promotes peer interaction. The (partial) anonymity provided by e-learning creates a secure environment that helps pupils overcome their fears while speaking a foreign language.

The transition to online training has also hastened the development of new abilities in both language students and educators, since many have been forced to navigate unfamiliar territory. English language instructors are using new abilities for the first time since the epidemic, including teaching online, making presentations online, providing students with online practice, utilizing electronic copies of coursebooks, and conducting online evaluations (Wright, 2021). Similarly, the virtual learning environment aided in the development of EFL students’ language skills, particularly their listening and speaking abilities ( Canals & Al-Rawashdeh, 2019), as well as their internet searching abilities ( Bernstein & Woosnam, 2019). Despite the challenges individuals had in adjusting to online settings, the new experiences stimulated the learning of unique talents and sharpened existing competence.

Similarly, the shift to digital learning has resulted in accidental learning ( *i.e.*, learning without intending to study) of English via casual exposure to the language. Because English is the most frequently used language online, accounting for 25.9 percent of all internet content, EFL learners are exposed to more English outside of their language sessions than they would in a face-to-face classroom. Additionally, claims that Saudi EFL students’ usage of the internet facilitates informal language learning by providing access to material that appeals to their interests, such as music, movies, YouTube videos, and videogames. Thus, internet education may be advantageous for students’ informal English learning.

Additionally, the need for unique abilities for online teaching and learning has resulted in a widespread demand for professional development opportunities. With the growth of COVID-19 cases and the tightening of quarantine rules, online training and instructional material have exploded in popularity. As a result, everyone now has access to a plethora of information through free internet webinars, conferences, courses, and workshops ( Jansen *et al.*, 2021). It has never been easier to attend educational and training activities in several locations without being restricted to a single location. Saudi universities all took part in these activities, granting admission to both university affiliates and non-affiliates. Additionally, some professors, educators, libraries, and even publishers made their resources freely accessible to guarantee that, even if the rest of the world grinds to a halt, education does not.

## Methods

The study used a mixed methods research design to collect data from the participants. On the quantitative part, a questionnaire was used, and semi-structured interviews with specific themes were used to collect teachers’ data. The former research paradigm allows for the researchers to demonstrate their survey to a large number of participants. So, the population will usually be represented thoroughly.

Two five-point Likert scale questionnaireswere designed by the researchers themselves. The first questionnaire was designed to check Saudi female students’ perceptions on the participation in LML. The students’ questionnaire consists of four domains: (1) students’ perceptions on LML, (2) students’ satisfaction for being indulged in such learning, (3) the difficulties that Saudi female students encountered and (4) the aids they received from others. The first domain includes 17 items, the second comprised six items, while the third has four items and the last domain includes five items. The questionnaire was sent to three ELT professors to referee its content validity. All their comments were considered. The teachers’ questionnaire includes only 11 items and grouped in one domain that is to examine their perceptions toward LML mode.

The participants of this part of the study comprised Saudi EFL female students studying for their bachelor’sdegree in English language and Translation at Qassim University, College of Language and Translation via the LML mode. They enrolled in different levels from one into four. Their median age is 23 years. The researchers shared the link of the digital questionnaire with all the female students. 101 responses were received and two of them were deleted because they were incomplete. All the ethical considerations were ascertained. Students were assured that they did not need to divulge their names or other personal information.

It is important to briefly mention how the authors performed the research ethics during this research. Beginning from the top to the bottom, the researchers got a consent letter (16-Eng-2021) from the committee of ethical issues in the college of Language and Translation at Qassim University. The consent letter was submitted to the head of the English department to follow-up the procedures including scheduling a time for the researchers to meet with the student and teachers to explain them the aim of the study. Likewise, all participants including 99 female students and 6 EFL teachers, were assured that their names are not required to be mentioned on the survey and were requested to select as precise alternatives as possible to assure the truthfulness of the study. Finally, the researchers promised them to share with them the finding of the study as soon as the study being completed. Students orally accepted to participate in the study and also agreed that their data would be published anonymous publicly.

The qualitative data were collected from the six EFL teachers that form the total faculty of EFL at the university where the study was conducted. The researchers isolated themes that have been shown by prior research to be predominant issues in EFL classrooms from the teachers’ point of view. Out of these, 11 themes relevant to the Saudi EFL context were picked to form the questions of the semi-structured interviews. The last question, numbered 12, was an open ended one which required teachers to list the positive and negative points of the LML practices in Saudi Arabia. Data from the 11 interview questions were quantified to establish leading trends.

## Data analysis and results

After collecting the students’ responses to the questionnaire over the period of a week, the data were analyzed using SPSS version 23. The data were coded in which the alternative strongly agree was coded into 5 and strongly disagree into 1. Both descriptive and inferential analyses were adopted.

Table 1 shows the Cronbach Alpha reliability test for the domains the study focused on. According to the table, all the domains got a strong to very strong reliability values except the instructors’ questionnaire which is considered as acceptable at (P= 0.69). The other domains, *i.e.*, students graded from good reliability as in the second and the third domain (P= .79) for both to very high as in the first domain where the reliability was shown as (P = .94).

**Table 1.** Reliability test of the variables.

Participants	Domain	Number of items	Reliability Cronbach Alpha
**Instructors**		11	0.69
**Students**	Perceptions	17.00	0.94
	Satisfactions	6.00	0.79
	Difficulties	4.00	0.79
	Aids	5.00	0.83

To answer the first research question (What perceptions do EFL instructors have about LML?),we turn to Table 2 which presents the Saudi EFL instructors’ views on LML. Six Saudi instructors provided their perceptions on their teaching experience using LML. According to the Table, instructors provided a moderate perception with a mean score and standard deviations of 3.59 and 0.44 respectively. The items show variations in levels of perceptions. A high perception in item 3 (4.17) reflected that instructors perceived the aids that LML provided them during their teaching experience. Yet, instructors showed low perceptions (2.33, 0. 82) as in item 11, which represented their dissatisfaction with the teaching experience while pursuing such kind of learning.

**Table 2.** Saudi EFL instructors' perceptions on LML.

Items	N	Mean	Std. Deviation	Relative importance %
1. In general, I am pleased with LML class	6	3.83	0.75	76.67
2. The course, which included both face-to-face mode and mouth-to-ear mode sessions, provided me with a beneficial teaching experience	6	4.67	0.52	93.33
3. This course aided my professional and personal growth	6	4.17	0.75	83.33
4. I am content with the degree of involvement in this course	6	3.67	1.03	73.33
5. In the future, I would be willing to teach in an LML mode	6	3.83	0.98	76.67
6. I am encountering many difficulties while teaching LML courses	6	2.83	0.98	56.67
7. My students experience many obstacles in LML course	6	3.00	0.89	60.00
8. LML has been greatly integrated into Saudi society	6	3.50	1.05	70.00
9. My teaching practices via LML has been amazing	6	4.33	0.82	86.67
10. My teaching practices via LML has been robotic	6	3.33	1.03	66.67
11. My teaching experience via LML has been dissatisfactory	6	2.33	0.82	46.67
**Total**	6	3.59	0.44	71.82

To answer research question 2 (What perceptions do Saudi EFL female students have about LML?), we turn to Table 3 which reflects Saudi EFL female students’ perceptions of LML. As the Table shows, 99 respondents answered to this category. They show a high mean score of (4.03,0.61) with a relative importance equal to 80.63 percent. Students’ perceptions were gathered viz-a-viz their responses to 17 items as Table 3 shows. Through all the items from 1 into 17, students showed high perceptions between 3.82 to 4.39, in which the highest perception was recorded in item 14, where students showed their enthusiasm towards such type of learning that they can learn without being put under stress. Gradually, students responded fall to the lowest point in (high level) where they scored (3.82, 0.87) as they showed their perceptions on their practice of English language in LML as they usually do in face-to-face mode.

**Table 3.** Saudi EFL female perceptions on LML.

Items	N	Mean	Std. Deviation	Relative importance %
1. The instructional resources on Lateral Multimodal Learning (LML) do an excellent job of explaining the English fundamental.	99	4.14	0.80	82.83
2. The study resources on LML were pertinent to the English course requirements.	99	4.08	0.78	81.62
3. LML was an extremely valuable supplement to the English course in terms of information and resources.	99	4.08	0.78	81.62
4. Using the study materials available on LML, I was able to get a thorough grasp of each lecture prior to attending.	99	3.82	0.87	76.36
5. I was able to revise more efficiently because of the learning tools on LML.	99	3.84	0.99	76.77
6. The learning resources on LML aided me in achieving a higher level of performance in assignments/course work.	99	4.09	0.86	81.82
7. LML has aided in the improvement of communication with the professor.	99	3.92	0.92	78.38
8. I believed that interactions with my colleagues in the LML forums aided me in comprehending the course information regarding the learning challenge of English.	99	3.96	0.91	79.19
9. Discussions in the forums aided my comprehension of the course material.	99	3.99	0.79	79.80
10. The quizzes and activities on LML were quite beneficial in aiding my comprehension of the English subject.	99	4.08	0.80	81.62
11. The feedback/answers I received on the tasks/quizzes in the English course were quite beneficial.	99	3.95	0.81	78.99
12. Studying via LML reduces the inhibition we have in using language in front of the instructor.	99	4.08	0.93	81.63
13. We practice English language the same as we do in face to face mode.	99	3.90	0.95	77.98
14. We feel more enthusiastic when we learnt without being put in stressful situations.	99	4.39	0.73	87.84
15. Teachers' explanations to the topics via LML go smoothly.	99	4.03	0.87	80.61
16. LML is compatible with Saudi culture, particularly among female.	99	4.11	0.87	82.22
17. As I see it, the LML course looks excellent.	99	4.08	0.87	81.62
**Total**	99	4.03	0.61	80.63

To answer the third research question (Are the Saudi EFL female students’ satisfied with their experience on LML?),we turn to Table 4 which presents Saudi EFL students’ perceptions on their satisfaction while learning through LML. As the Table exhibits, students perceived the LML learning mode satisfactorily, they scored a total mean score of 3.88 with a standard deviation of 0.67, which is considered as high according to the statistical measures. Their satisfaction regarding their performance to the course requirements (item 18) and their present progress (item 19) scored high with mean scores 4.01 and 4.02 respectively. Despite the high level of satisfaction as calculated in the total mean scores, items 21 and 23 scored moderately, where their mean scores and standard deviations were (3.56, 1.00 & 3.58, 0.99) respectively. They showed moderate perceptions regarding the progress they achieved in the language skills and contents attainted within the course they studied.

To answer the fourth research question (Do Saudi EFL female students face difficulties while learning through LML?), we see that Table 5 presents Saudi EFL female students' perceptions on the difficulties they faced while appearing in LML. Students showed that they face difficulties in learning though LML, such difficulties rated as moderate though. 68 of them (M = 3.39) agreed that they face difficulties on LML learning mode as a result of missing the facial expression of the instructors or instructors’ dominance in lecturing or the difficulties stem from the robotic or mechanical feeling they got while learning when the instructors are not seen or interacted with. To answer the last research question, Do Saudi EFL female students get help from other while learning though LML. Table 6 represents Saudi EFL Saudi female students’ perceptions on the source of aids they use integrated with LML learning. According to Table 6, students showed high perceptions on the aid they got with a total mean score of (4.02) and a standard deviation of (0.78). The aids gradually rated from the family as high as (M = 4.31, Std. = 0.74) to the aids they got from the college administration which scored as (M = 3.83, Std. = 0.99).

**Table 4.** Saudi EFL female students' satisfactions on learning though LML.

Items	N	Mean	Std. Deviation	Relative importance %
18. I am very satisfied with my performance in achieving the course requirements.	99	4.01	0.98	80.20
19. I am satisfied with my present progress.	99	4.02	0.96	80.40
20. I feel LML delivery mode restricts our integrity as females in the society.	99	3.70	1.02	74.08
21. I believe I made progress in the skill-based courses only.	99	3.56	1.00	71.22
22. I believe I made progress in language functions.	99	3.95	0.81	78.99
23. I believe I made progress in the content-based courses only.	99	3.58	0.99	71.52
**Total**	99	3.81	0.67	76.10

**Table 5.** Saudi EFL female students' perceptions on the difficulties they face in LML learning.

Items	N	Mean	Std. Deviation	Relative importance %
24. I have not faced anything that impeded my ability to learn effectively.	99	3.85	1.03	76.97
25. Missing the instructor's facial expression impeded my ability to learn effectively.	99	3.18	1.42	63.64
26. Teachers' dominance in such learning impeded my ability to learn effectively.	99	3.24	1.33	64.85
27. LML is a robotic learning which decreases our motivation to learn.	99	3.30	1.32	66.06
**Total**	99	3.39	1.01	67.88

**Table 6.** Saudi EFL female students' perceptions of the aid they got while indulging in LML.

Items	N	Mean	Std. Deviation	Relative importance %
28. The greater liberty that we get in LML has aided my studying in this course the most.	99	4.01	0.97	80.21
29. Despite the less contact with the instructors, LML has aided my studying in this course the most.	99	4.01	0.99	80.20
30. The college administration has aided my studying in this LML course the most.	99	3.83	0.99	76.57
31. My family has aided my studying in this LML course the most.	99	4.31	0.74	86.12
32. I prefer LML mode over the face to face delivery.	99	93.94	1.28	78.79
Total	99	4.02	0.78	80.36

This study hypothesizes that there are significant correlations between the four domains at a point of 0.05.

Table 7 presents the correlation between the four domains, which are, students’ perceptions, satisfactions, difficulties, and aids. Pearson correlations coefficient was used to show the relationships amongst the previously mentioned domains. The findings show that there are correlations between students’ perception and satisfaction (r = .719) regarding the studying through LML mode, students’ perceptions and aids (r = .659.) Still there is a correlation between students’ perceptions and difficulties that face, such correlation is not available, not strong though, (r = .351). Table 7 also presents the correlation between the second domains, *i.e.*, students’ satisfactions with the difficulties and the aids they got while learning through LML mode, (r = .582, r = .656) respectively. The third domain, *i.e.*, students’ difficulties were also correlated with the aid they got, yet that correlation is not significant (r = .261).

**Table 7.** Pearson Correlation Coefficient among the four domains.

Domain		1	2	3	4
1	Pearson Correlation		.719 ^**^	.351 ^**^	.659 ^**^
	Sig. (2-tailed)			.000	.000
2	Pearson Correlation			.582 ^**^	.656 ^**^
	Sig. (2-tailed)			.000	.000
3	Pearson Correlation				.261 ^**^
	Sig. (2-tailed)				.009
4	Pearson Correlation				
	Sig. (2-tailed)				

The qualitative data collected from the teachers through the semi-structured telephonic interviews established clear trends in their perceptions of LML. The results are quantified in Table 2.

Teachers’ responses show that the Saudi academia is ready to follow the current method of LML, which is a happy situation for all stakeholders including administration, funding agencies, teachers and learners who have become well adapted to the mode in use in the last two years. It is notable that teachers perceive this as ranking high on the professional satisfaction scale, with as much as 93.33% relative importance being given to LML on the benefits accrued to teaching experience. Professional growth (83.33%), LML for future teaching plans (76.67%), and teachers’ degree of involvement while using LML (73.33%) are encouraging outcomes. On the flip side, difficulties in the teaching experience (56.67%), obstacles in delivering the LML course pack (60.0%), and the mechanical nature of the experience (66.67%) are the areas that need to be checked in the future.

Finally, responding to the open-ended question that elicited responses on the positive and negative aspects of the practice, teachers listed some challenges such as learning how to fulfill the demands of the new mode, making the classes interesting to ensure learner engagement, and using annotations as support materials for the main teaching. Two of the teachers interviewed also pointed out that the nature of communication in M2E is abstract which is sometimes a challenge for both teachers and learners, though they agreed that this could be a fallout of the general psychological ramifications of the pandemic-imposed restrictions, and not necessarily attributable to the M2E mode of learning. Apart from this, one teacher felt that knowledge transfer was incomplete in M2E as the entire gamut of paralinguistic communication was shelved in this method with learners being totally invisible to the teacher. On the other hand, the benefits were more robust, including the usefulness of the method in developing learners’ oral communication, presentation skills, and listening skills.

## Discussion

This study found that Saudi EFL students perceived LML in moderate level. This finding can be interpreted to indicate the success of the support that the Kingdom of Saudi Arabia supplied to the virtual learning initiative, though this type of learning does not yet reach the level of conventional learning due to many reasons, say the long of time needed to prepare virtual learning ( Jansen *et al.*, 2021), or the non-readiness of the organization to cope up with the transfer ( Hazaea *et al.*, 2021). The transition to online education has brought to light numerous additional challenges impeding students’ advancement. One of the issues identified is a lack of digital ready skills among students which is exacerbated by a lack of technological assistance. Additionally, students spend considerable effort resolving such challenges and traversing online territory.

The findings of this study further showed that Saudi EFL female students have high positive perceptions on studying through LML. This finding opens our expectations to think about the future of virtual learning in the Saudi context. This finding is in line with Villegas *et al.* (2020) who found that Saudi students positively view virtual learning as such kind of learning break down their shyness which usually associated with conventional classrooms.

Remote learning enables students to connect to their virtual classes from any location. However, distant learning’s adaptability is a double-edged sword. On the one hand, it reduces physical constraints such as location and time, enabling access to learning and development opportunities around the globe and reducing travel times to and from university campuses. On the other side, it provides for more distraction from educational activities. The home setting is fraught with disturbances, especially when additional family members work and study from home. Additionally, the lack of separation between the home and school settings makes it more difficult for students to differentiate between leisure and academic time.

Furthermore, EFL Saudi female students expressed high positive satisfactions towards LML learning mode. This finding can be interpreted socio-culturally. Female students living in a conservative society and guided by the Islamic creeds finding their target in such kinds of learning and without being put in any embarrassing situations arising from co-education or even with facing instructors.

This study also explored the difficulties that female students encounter in indulging in LML. The students reported that face difficulties in a moderate level. These difficulties may stem from the missing of instructors’ facial expression which may help in understanding the content. LML learning is different from virtual learning that no-one-sees-the-others. Other may see this learning as a robotic learning where no real interaction occurred. Students need visual input of spoken language, primarily for language development. Not using video elements throughout the class may jeopardize the students’ development of speaking and listening abilities. Alternatively, students’ vocabulary and research abilities may develop as a result of incidental learning that happens as a result of browsing the internet and all its English information. Nonetheless, testing the development of these language abilities online has shown to be a challenge that needs instructors to take additional care to ensure the validity of the assessment.

Students reported highly positive attitudes that they got help from others which help them in pursing this learning most, the highest help was supplied by their family, in addition to the college administration. It is the secret behind the high positive perceptions that students reported earlier. The study found that there is good correlation between students’ perceptions and satisfaction in this learning more (r = .72) and perceptions and the aids they got (r = .66).

Teachers, though only a small number could be interviewed given the limited faculty size in the university, appeared upbeat about the LML mode of teaching-learning and expressed readiness to continue with the mode in the long run.

## Significance of the Study

The significance of this study stems from two axes. Firstly, the reach and unavoidability of the online educational mode in EFL in the coming future with new waves of the pandemic being forecasted. Secondly, the goals of Saudi Vision 2030 clearly state the desire of the administration to produce a generation of educated young people who can exploit the vast potential of the global job market. In this background, and of the so far subdued socio-economic status of Saudi women, this study is vastly significant.

## Conclusion

This study reported the perceptions of Saudi EFL instructors and students toward a new mode of learning called LML. They study reported medium preference of instructors to such kind of leanings. They also showed some points which needs to be worked out to efface this learning mode, like how to deliver the content of the course effectively and attaining students’ motivations. Furthermore, the study explored four domains in Saudi EFL female students, *i.e.*, perceptions, satisfaction, difficulties and aids. The study reported good correlations between perceptions and satisfaction and aids. It also reported good correlations between satisfaction and aids. In conclusion, educators and stakeholders must examine the aforementioned challenges while assessing the effectiveness of e-learning in EFL and assessing its use and applicability after the epidemic. Future study in this area should examine the disparity in learner motivation between those who are more likely to engage in online peer conversations and those who are less motivated to participate when courses are conducted online. Additionally, larger-scale research is required to determine the academic EFL learning results at the university level in Saudi Arabia.

This research may be expanded to include the perspectives, opinions, and impressions of educational institution stakeholders, such as ELT department chairs, curriculum designers, and publishers, as well as the students themselves. The research may always be replicated in another EFL setting using identical parameters. Nevertheless, the current study has significant shortcomings that should be addressed in future research. Qualitative components such as group discussions and interviews might be added to the quantitative data to bolster them. The efficiency of online learning through Blackboard was determined by a poll of students’ views, not actual learning results. This may have been accomplished more effectively by assessing learning outcomes and comparing them to those of a control group that did not use Blackboard for instruction. Additionally, the research was limited to a single institution; if data were gathered from several Saudi universities, broader conclusions may have been drawn.

## Recommendations

Whereas the current study concluded that female EFL students in Saudi Arabia perceive online language learning positively, this finding contradicts many other previous studies like Macalister and Nation (2019), Prakash and Murthy (2019), and Raygan and Moradkhani (2020). Such studies reported that virtual leaning demotivates learners. This contradiction is important and should be diligently reinvestigated in future studies. It can be justified to the context of the participants and their ethical and religious heritage. Furthermore, the studies reported herein have been conducted at the climax of the pandemic, now after the cease of the curve, the situation becomes better and both students and instructors accustomed and be familiar with virtual learning. Therefore, it is also recommended that before generalizing the results of this study, deeper investigations into other online learning environments and conditions in EFL should also be investigated. Similarly, the number of teachers enrolled in the study was small at six, though it was a condition beyond the researchers’ control, future studies may be undertaken with a larger teacher base.

## Limitations

The gender specificity of this study was a limitation but one which could not be overcome due to the sociocultural milieu of Saudi Arabia. Another limitation was the wholly quantitative data and that too, to the exclusion of the EFL teachers’ point of view.

## Data availability

Face-to-face and mouth-to-ear modes - https://doi.org/10.6084/m9.figshare.18667568.v1.

Questionnaire for Instructors.csv - https://doi.org/10.6084/m9.figshare.18667571.v1.

Students' Questionnaire.csv - https://doi.org/10.6084/m9.figshare.18667574.v1.

Questionnaire for Instructors - https://doi.org/10.6084/m9.figshare.18667577.v1.

Data are available under the terms of the Creative Commons Attribution 4.0 International license (CC-BY 4.0).
