# Supplementary material for: Lateral Multimodal Learning in a Saudi EFL Context: Investigating the Perceptions of Teachers and M2E Female Learners
Source: F1000Res. 2022 Mar 30;11:369. [Version 1] doi: 10.12688/f1000research.109454.1 (PMC9034168; doi:10.12688/f1000research.109454.1)
Supplement: Peer review history

# Peer review: Reviewer response for version 1

**DOI:** 10.5256/f1000research.120953.r138925

**Published:** 2022-05-30

**Authors:** Wafa’ A. Hazaymeh

**Affiliations:** 1 Al Ain University, Emiratos Árabes Unidos, United Arab Emirates

A well-written article with well-presented and arranged topics. It focuses on an up-to-date field of Applied Linguistics. The topic selected is current and vital, and the writers' writing style is engaging and exceptional. The analysis is precise and critical. It reflects the writers' deep grasp and well-presented argument. In overall, the essay gives correct discussion and derives new and significant discoveries with future implications. The writers have also addressed the research questions correctly and in a very interesting and transparent manner. Furthermore, there are no grammatical, punctuation, spelling, or typographical errors in the document.

 However, please be careful with the following points:

  Certain references should be changed to include the DOI of each reference of the following:

1. Al-Ahdal AAMH, Alqasham FH: Saudi EFL learning and assessment in times of covid-19: Crisis and beyond. *Asian EFL Journal.* 2020a; **27**(4.3): 356–383.
2.  Al-Ahdal AAMH, Alqasham FH: WhatsApp in language classroom: Gauging Saudi EFL teachers' roles and experiences. *Opcion.* 2020b; **36**: 1667–1680.
3.  Al-Ahdal AAMH, Hussein NMA: WhatsApp as a writing tool in EFL classroom: An study across two universities in Saudi Arabia. *Asian EFL Journal.* 2020; **27**(3.1): 374–392.
4. Hazaea AN, Bin-Hady WRA, Toujani MM: Emergency remote English language teaching in the Arab league countries: Challenges and remedies. Computer-Assisted Language Learning Electronic Journal. 2021; 22(4): 201–222.

Moreover, some references are not highlighted in the research as the rest of the references such as:

1. Al-Ahdal& Alqasham, 2020
2. Mackay 2019
3. Wright, 2021

In addition, there should be a space between:

  

- **bachelor’s degree**
- (What perceptions do EFL instructors have about LML?) **, we**. Please use **the researchers** instead of we in the article

I certify that I have read this work and have the requisite level of expertise to ensure that it fulfills an adequate research standard.

Is the work clearly and accurately presented and does it cite the current literature?

Yes

If applicable, is the statistical analysis and its interpretation appropriate?

Yes

Are all the source data underlying the results available to ensure full reproducibility?

Yes

Is the study design appropriate and is the work technically sound?

Yes

Are the conclusions drawn adequately supported by the results?

Yes

Are sufficient details of methods and analysis provided to allow replication by others?

Yes

Reviewer Expertise:

NA

I confirm that I have read this submission and believe that I have an appropriate level of expertise to confirm that it is of an acceptable scientific standard.

# Peer review: Reviewer response for version 1

**DOI:** 10.5256/f1000research.120953.r129325

**Published:** 2022-04-07

**Authors:** Prof. Abdul-Hafeed Ali Fakih

**Affiliations:** 1 Department of English, Najran University, Najran, Saudi Arabia

The manuscript is well-written, and ideas are outstandingly presented and greatly organised. It discusses an under-researched area of Applied Linguistics: Lateral Multimodal Learning. The topic identified is relevant, significant, and the authors' style of writing is engaging and impressive. The analysis is very fit and critical. It displays the authors' thorough understanding and carefully presented argument. The manuscript, in a nutshell, presents, compares/contrasts perspectives, and draws original and meaningful conclusions with future implications. The authors have also managed to satisfactorily answer the research questions in a very interesting and lucid manner. That apart, the manuscript is also free from grammar, punctuation, spelling errors, and typos.

 There are, however, two issues that need to be rectified, as pointed out below:

1. The expression Mouth-to-ear (M2F) should be given in full in the title. I find the coinage really appealing. Kudos!
2. In lines 3 and 4 of "Academic institutions in KSA", the in-text citation (Canals & Al-Rawashdeh, 2019) should be replaced with (Alqahtani, 2022), considering the publication date and appropriacy of the reference. Thus, in the reading list, too, the following reference (a, below) should be used in place of b.
  a. Alqahtani, M. H. (2022). Post pandemic era: English language teachers' perspectives on using the Madrasati e-learning platform in Saudi Arabian secondary and intermediate schools. World Journal of English Language, 12(2), 102-116. ^1^
  b. Canals L, Al-Rawashdeh A: Teacher training and teachers’ attitudes towards educational technology in the deployment of online English language courses in Jordan. Computer Assisted Language Learning. 2019;32(7):639-66

Is the work clearly and accurately presented and does it cite the current literature?

Yes

If applicable, is the statistical analysis and its interpretation appropriate?

Yes

Are all the source data underlying the results available to ensure full reproducibility?

Yes

Is the study design appropriate and is the work technically sound?

Yes

Are the conclusions drawn adequately supported by the results?

Yes

Are sufficient details of methods and analysis provided to allow replication by others?

Yes

Reviewer Expertise:

NA

I confirm that I have read this submission and believe that I have an appropriate level of expertise to confirm that it is of an acceptable scientific standard.

## References

[1] : Post pandemic Era: English Language Teachers‟ Perspectives on Using the Madrasati E-Learning Platform in Saudi Arabian Secondary andIntermediate Schools. *World Journal of English Language* .2022;12(2) :102-116 Reference source

# Response

**Published:** 2022-04-07

**Authors:** Arif Ahmed Mohammed Hassan Al-Ahdal

**Affiliations:** Department of English Language, College of Arts and Sciences, Saudi Arabia

Thank you so very much for the encouraging words and  insightful comments, Prof. That, indeed,  makes great sense. Thanks heaps once again!
